# Management and outcome of benign acute childhood myositis in pediatric emergency department

**DOI:** 10.1186/s13052-021-01002-x

**Published:** 2021-03-09

**Authors:** Giacomo Brisca, Marcello Mariani, Daniela Pirlo, Marta Romanengo, Angela Pistorio, Alberto Gaiero, Chiara Panicucci, Emanuela Piccotti, Claudio Bruno

**Affiliations:** 1 Subintensive Care Unit, IRCCS Istituto Giannina Gaslini, via Gerolamo Gaslini 5, 16147 Genoa, Italy; 2 Infectious Disease Unit, IRCCS Istituto Giannina Gaslini, Genoa, Italy; 3 Department of Epidemiology and Biostatistics, IRCCS Istituto Giannina Gaslini, Genoa, Italy; 4 Pediatric and Neonatology Department, ASL2 Savonese, Savona, Italy; 5 Center of Translational and Experimental Myology, IRCCS Istituto Giannina Gaslini, Genoa, Italy; 6 Pediatric Emergency Unit, IRCCS Istituto Giannina Gaslini, Genoa, Italy

**Keywords:** Children, Creatine kinase, Rhabdomyolysis, Gait abnormalities, Clinical pathway

## Abstract

**Background:**

Benign acute childhood myositis (BACM) is a self-limited syndrome associated with viral infections characterized by symmetric lower extremity pain typically affecting school-aged children. Evolution in rhabdomyolysis and kidney damage is rarely reported.

Despite this, the acute presentation commonly concerns both parents and health care providers, often leading to unnecessary workup.

The aim of the study was to determine the features and outcome of a large series of children with BACM identifying a management pathway for pediatricians in Emergency Department (ED).

**Methods:**

We conducted a retrospective study of patients with BACM managed in 2 Italian pediatric ED during a period of 8 and a half years.

Demographic data, clinical, and laboratory results were extracted from electronic medical records.

Recurrence, complications, treatments, and outcomes were also recorded.

Descriptive statistics were produced for first-episode patients and for those with recurrence of myositis. A comparison between groups was performed.

**Results:**

One hundred and thirteen patients with BACM were identified. Ninety-two children (65 males) had a single episode, while ten (nine males) had recurrence. The mean age at presentation was 6.0 years (range 2–13,2).

All patients had normal neurological examination and no one developed myoglobinuria, or renal failure. At first evaluation median CK level was 1413 UI/l (normal values < 150 U/L).

Median CK of “recurrent” patients was higher than “non-recurrent” (2330 vs 1150 U/L, *p* = 0.009).

Viral studies were positive in 51/74 cases, with high prevalence of Influenza viruses.

Ninety-six patients (85%) were hospitalized with a median of 4 days. No patients had any residual muscular impairment.

**Conclusions:**

BACM has an excellent prognosis. Severe pathological conditions can be excluded with a complete history and clinical examination and simple blood and urine tests, avoiding unnecessary diagnostic investigations. Most patients may be discharged home from ED recommending hydration, rest, analgesics and careful follow-up.

## Introduction

Benign acute childhood myositis (BACM) is a rare, transient, self-limiting syndrome, affecting mid school children (usually males). It is characterized by prodromal viral illness followed by calf tenderness or pain and sudden walking abnormalities which occur on average 3 days as the initial viral illness resolves [1]. Muscle pain usually affects the gastrocnemius and soleus group with symmetric distribution and is associated with rise in serum level of muscle enzyme, including serum creatine kinase (CK). The hallmark of BACM is spontaneous clinical resolution within 1 week. Nevertheless, evolution in rhabdomyolysis, and kidney damage has been rarely reported [2].

BACM can occur sporadically or in epidemics. Several authors have confirmed the association with I*nfluenza B* [3] and other viruses, including *Influenza A, Parainfluenza, Adenovirus, Coxsackievirus, and Mycoplasma pneumoniae* [4–6]. Recurrence of BACM in the same individual has been occasionally described [1].

Since this condition is characterized by benign prognosis and short duration of symptoms, few efforts have been applied in order to define the pathogenesis of BACM. Electromyograms recorded during BACM episodes resulted normal or with patchy myopathic changes [7]. Few muscle biopsies collected from patients affected from BACM showed normal morphology [7, 8] or demonstrated segmental rhabdomyolysis [1] or myositis features, such as moderate muscle necrosis with interstitial inflammation [9–11]. Moreover, few reports focused on muscle MRI findings in BACM patients, showing aspecific signal abnormality in gastrocnemius and soleus muscles [12].

Despite its benignity, BACMs can be frightening to parents and confusing to physicians who are not familiar with this entity, leading to unnecessary extensive workups.

In the present study, we retrospectively reviewed data from children who presented with BACM at two pediatric Emergency Departments (ED) in order to analyze the main clinical, laboratory and etiological features, to evaluate morbidity and outcome, and to design a management pathway.

## Methods

We retrospectively reviewed data from all children diagnosed with BACM between January 1, 2010 and June 31, 2018, at two different pediatric ED, from Gaslini Children’s Hospital (Genoa, Italy) and from San Paolo Hospital (Savona, Italy).

Demographic and clinical characteristics including age, sex, clinical presentations, history of fever or any other symptoms, occurrence of myoglobinuria, therapeutic management, outcome and laboratory reports were extracted from electronic medical records.

In all patients blood examination including complete blood count, serum CK and renal function assessment, as well as urine dipstick test, was performed.

Seventy-four patients (66%) were tested for viral infections. Of these, 33 were tested by polymerase chain reaction (PCR) on throat swab, 31 by serology, and 10 by both tests.

Descriptive statistics were produced for demographic, clinical and laboratory characteristics of patients. Mean and standard deviation (SD) are presented for normally distributed continuous variables, median and interquartile ranges (IQR) for non-normally distributed. Numbers and percentages were used for categorical variables.

To compare groups, for continuous variables, parametric (t-test) or non-parametric (Mann-Whitney or Kruskal Wallis when appropriate) tests were performed according to data distribution.

## Results

### Demographic data

One hundred and thirteen patients with a diagnosis of BACM were identified. Main patients’ demographic, clinical and laboratory features are summarized in Table 1.

**Table 1:** main features of patients with BACM

Total BACM episodes, n	113
**Patients with at least one BACM, n**	102
- Patients with single BACM, n, %	92 (90%)
- Patients with recurrent BACM, n, %	10 (10%)
**Sex:** male, n (%), female, n (%)	74 (73%), 28 (27%)
**Age:** median, range	6,0 years, 2,0–13,2 years
**Fever:** n (%)	96 (85%)
**Muscle pain:** n (%)	103 (91%)
**Gait abnormalities:** n (%)	87 (77%)
- Refuse to bear weight	17 (15%)
- Walking on toes	12 (11%)
- Wide base gait	9 (8%)
- Motor embarrassment/difficulties	49 (43%)
**Creatine kinase:** median (range)	1413 IU/L (257–12,858 IU/L)
**White cell count:** median (range)	4650/μL (1950–11,980)
- **Neutrophils**	2005/μL (372–8400)
- **Lymphocyte**	1904/μL (340–6800)
**C reactive protein:** median (range)	0 mg/dl (0–2,65)
**Viral studies:** n (%)	74 (65%)
**Viral studies positive:** n (%)	51 (45%)
- Influenza B	22 (19%)
- Influenza A	15 (13%)
- Coxsackievirus	7 (6%)
- Adenovirus	4 (4%)
- Echovirus	2 (2%)
- Mycoplasma	1 (1%)
**Hospitalization: n (%)**	96 (85%)
**Median length of stay:** days, range	4 days, 1–10
**Myoglobinuria/Acute renal failure**	0
**Return ED visit**	0

Ninety-two children (65 males and 27 females) had a single episode of BACM, nine (eight males and one female) had a second episode and one boy presented with three episodes.

**Fig. 1:**
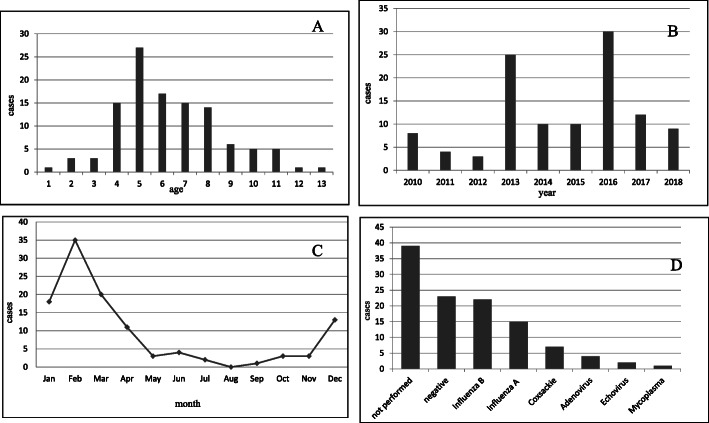
Case distribution according to age (1 A), year (1 B) and month of presentation (1 C). Microbiological results in children with benign acute common myositis (1D)

The median age at presentation was 6.0 years (range 2–13.2 years) **(**Fig. 1**a).**

Overall, 50% of total BACM episodes were registered in 2013 and 2016 years and most episodes (eighty cases, 71%) occurred in winter months with only seven cases in summer **(**Fig. 1b and c**).**

### Clinical features

Fever at presentation or in the week before was detected in 85% of the cases.

Bilateral muscle pain predominantly confined to the calves muscles was reported in 104 out of 113 episodes (92%).

At first clinical examination in ED, gait abnormalities were registered in 87 cases (77%): in particular, seventeen children (15%) completely refuse to bear weight and twelve patients (11%) walked on their toes. Nine patients (8%) presented with wide-based gait while in forty-nine cases (43%) gait abnormalities were generally defined as “motor embarrassment/difficulties”.

Muscle strength, tone, and osteotendinous reflexes of the lower extremities were always conserved.

Clinical examination also showed signs and symptoms of respiratory tract infection (39%), vomiting and/or diarrhea (8%), skin rash on the trunk (6%).

No patient reported to have dark urine emission nor facial cutaneous involvement.

Blood pressure was normal for age in all patients.

### Laboratory findings

At presentation median serum CK was 1413 U/l (normal values < 150 U/L, range 257–12,858 U/L); median absolute leukocyte count was 4650 elements/μl, and 27% of the patients showed neutropenia (< 1500/μl). Hemoglobin, platelets count, urea blood nitrogen and creatinine level were within normal values in all patients. Urine dipstick test was negative in all patients, and therefore urine microscopy was not performed.

Viral studies were positive in 51/74 cases (69%), with a high prevalence of *Influenza* viruses (37 cases) followed by *Coxsackievirus* (7 cases), *Adenovirus* (4 cases), *Echovirus* (2 cases) and *Mycoplasma pneumoniae* (1 case)**.** Within the *Influenza* virus, the *Influenza B* was detected in 22 cases, and the *Influenza A* in 15 cases **(**Fig. 1**d)**.

Median CK of patients with influenza-associated BACM was similar to patients with other virus-associated BACM and virus negative patients (*p* = 0.24).

### Outcome

Ninety-six patients (85%) were hospitalized and treated with intravenous hydration and oral analgesics as necessary.

During the hospitalization myoglobinuria never occurred in any patient and no renal/hydro electrolytic abnormalities were observed.

Mean duration of hospitalization was 4 days. Length of stay was similar among patients with influenza-associated BACM and other patients’ groups (*p* = 0.89)**.**

Seventeen patients (15%) were directly discharged from ED and referred to the family pediatrician for following clinical evaluation and biochemical follow-up with serum CK level dosage.

Oral analgesics, rest and adequate amount of oral liquids assumption were recommended at home. All children revealed clinical and laboratory improvement. No patients had any residual muscular impairment nor other complications and there was no ED return in the following month.

Median age and CK level were similar for discharged and hospitalized patients (6 vs 5.9 years, 1413 vs 1413,5 IU/L).

### Recurrent patients

Nine patients (8 M/1 F) presented with a second episode of BACM between 6 months to 5 years after the first episode, and one patient had three episodes within three years.

The main characteristics of “recurrent” patients are summarized in Table 2.

**Table 2:** key features of patients with recurrent BACM

Patient	Sex	Episode of BACM	Date of visit	Age (years)	Virus	Creatine-kinase (IU/L)
**1**	**male**	**1**	18/01/13	5	Influenza B	5835
		**2**	03/02/18	10	n.d.	1586
**2**	**male**	**1**	14/02/16	3,3	Influenza A	2861
		**2**	14/12/16	4,2	Coxsackie	467
**3**	**male**	**1**	18/03/13	3,1	Influenza A	1674
		**2**	30/01/15	4,9	Influenza B	2710
**4**	**male**	**1**	30/04/15	6,7	Influenza B	1153
		**2**	12/04/18	9,6	Influenza A	2330
**5**	**male**	**1**	20/01/18	4,4	n.d.	1920
		**2**	25/02/17	3,5	n.d.	4457
**6**	**female**	**1**	27/02/16	8,1	Influenza B	3084
		**2**	07/04/15	7,25	Influenza B	1670
**7**	**male**	**1**	17/06/15	4,8	n.d.	7450
		**2**	26/12/15	5,3	n.d.	4166
		**3**	15/01/17	6,3	n.d.	5630
**8**	**male**	**12**	02/12/17	6,9	Coxsackie	1698
		**12**	19/03/15	4,2	Influenza B	1137
**9**	**male**	**21**	16/02/14	4,4	n.d.	2200
		**12**	21/02/15	5,4	n.d.	481
**10**	**male**	**21**	04/03/16	5,1	Influenza B	7804
		**32**	24/02/18	6	Influenza A	6005

**Fig. 2:**
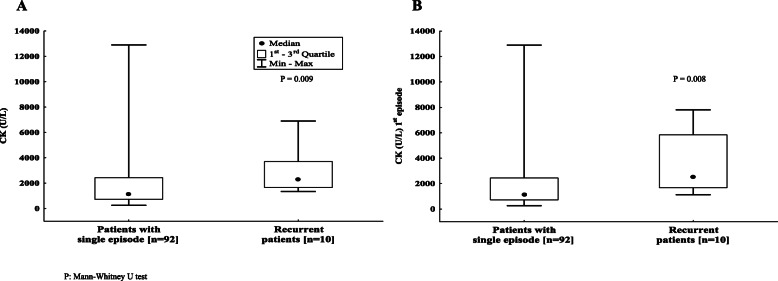
CK levels in patients with single and recurrent benign acute common myositis: despite a great variability in CK levels for patients with single episode, median CK is statistically lower than recurrent patients (2 A). Similar results were found considering only the first episode for recurrent patients (2 B)

At presentation, their median serum CK was higher than those “non-recurrent” (2330 vs 1150 U/L, p = 0.009) **(**Fig. 2**a),** even considering only the first episode (2265 vs 1150 U/l U/L; *p* = 0.008) **(**Fig. 2**b).**

In 13 out of 21 total episodes microbiological tests identified a viral infection, with large prevalence of *Influenza* viruses (11 cases). In four patients, distinct episodes were associated with two different viruses, while Patient 6, the only female, presented two episodes within ten months both associated with *Influenza B* virus.

## Discussion

In last decades many case series of BACM have been reported. Middleton et al. in 1970 showed a strong association with *influenza B* infection [13] and subsequent studies have confirmed the epidemiologic association with viral infection and the benign course of the disease [3, 4, 14, 15].

However, the acute presentation commonly concerns both parents and health care providers, often leading to unnecessary workup.

This is the largest series reporting on clinical course of BACM in children, and our findings strongly support the benign course of this condition.

All patients presented with the classic clinical picture characterized by febrile prodromes, followed by acute onset of symmetrical calf muscle pain and abnormal gait.

At first examination, 19% of patients completely refused to load weight on their legs, while a large proportion was described with a general motor embarrassment. This clinical presentation resembles the “Frankenstein gate”, a wide based, stiff lagged gait, largely observed in BACM series [16].

Beyond the gait difficulties, all patients presented with normal neurological examination, a clinical turning point in the differential diagnosis of patients presenting with acute onset of gait abnormalities.

Table 3 shows the main causes of gait abnormalities and/or acute legs pain in children in differential diagnosis with BACM. Among them, Guillain Barrè syndrome (GBS) needs to be promptly recognized, given the possibility of setting up a timely therapy. This is particularly true for younger patients who are unable by age to clearly express symptoms, with mild serum CK increase and even if the osteotendon reflexes are preserved [17]. In these cases, a neurophysiological study should be considered.

**Table 3:** differential diagnosis of BACM

Guillain-Barrè syndrome	Symptoms onset 2–4 weeks after viral illness
	Distal paresthesiaand ascending paralysis
	Symmetric weakness with absent/decreased/preserved deep tendon reflexes
	Normal or slightly increased serum CK
**Dermatomyositis**	Subtle onset and chronic course
	Proximal muscle weakness
	Skin involvement
**Muscular dystrophy**	Muscle weakness
	Chronic persistent increase of CK levels
	Possible family history of neuromuscular conditions
**Juvenile Idiopathic arthritis**	Asymmetric distribution with swelling and tenderness in joints
	Subacute onset
	Normal CK levels
**Transient synovitis of the hip**	Symptoms onset 2–3 weeks after viral illness
	Asymmetric pain and limited motion of the hip
	Normal CK levels
**Osteomyelitis**	Frequent hystory of trauma or penetration of the skin
	Elevation of inflammatory markers
	Swelling of soft tissues in affected area with or without erythema
**Myalgia associated to Influenza**	Less severe
	Concomitant with viral symptoms
	Normal CK levels

In the pediatric age, viral myositis is the most common recognized cause of rhabdomyolysis [18], and the most dangerous sequela of rhabdomyolysis with myoglobinuria is acute renal failure. Data on pediatric population are limited and predictive factors to determine the evolution to acute renal failure are still lacking.

However, in our series, even cases with massive CK increase did not present myoglobinuria nor acute renal failure, supporting that BACM has a benign clinical evolution with an excellent prognosis.

Mannix and colleagues demonstrated that patients with rhabdomyolysis with urine dipstick negative for heme are at a much lower risk of developing acute renal failure, pointing out urinary dipstick as a cheap and not invasive screen test for identification of patients who need renal function monitoring [18].

Considering that, routinary blood testing, including serum CK and renal function and dipstick urinalysis are sufficient and children with BACM can be managed as outpatient with analgesia, rest and appropriate clinical and laboratory follow-up.

BACM can occur sporadically or in epidemics; since the first report by Middleton and colleagues [13], several authors have confirmed the association with *Influenza virus A* and *B*; however other viruses, including *parainfluenza, Adenovirus, Coxsackie* and also *Mycoplasma pneumoniae* have been isolated [19].

Our large series confirms the strong association between BACM and influenza viruses, highlighting the major role of Influenza B virus. However, the mechanisms by which a viral infection leads to muscle involvement are poorly understood.

In our study BACMs associated with influenza or other viruses showed no clinical differences. In particular, median CK at presentation and median hospitalizations were similar among the groups.

Recurrence of BACM has been previously reported in some series of patients but no specific details are provided. In our series we documented a rate of recurrence of 9.8%, similarly to other reports [1].

Unlike other authors we also found one patient who experienced three episodes of BACM in the range of three years.

Ruff and Secrist [9] proposed that BACM in mid-childhood would appear at the first exposure to a specific influenza virus (thus explaining the few cases reported in adults) and that recurrence of BACM would be induced only by other virus infection.

However, patient 6, had two different events of BACM caused by two documented infections with Influenza B virus.

As supported by our data, it is known that during influenza epidemics only a small proportion of children develop BACM, that they are mostly males and that the involvement of siblings and recurrent patients have been reported [3, 4].

Moreover, we found that CK level of recurrent patients at presentation were significantly higher than non-recurrent patients.

This raises the hypothesis that a genetic susceptibility might sustain a metabolic impairment in the skeletal muscle tissue which is triggered by a viral infection, similarly to metabolic myopathies. Further research is needed to better understand the pathogenesis of recurrent BACM.

Our study is limited by its retrospective nature. Moreover, for our data analysis we had to rely on information reported by pediatricians on electronic medical records, which might be incomplete.

## Conclusions

**Fig. 3:**
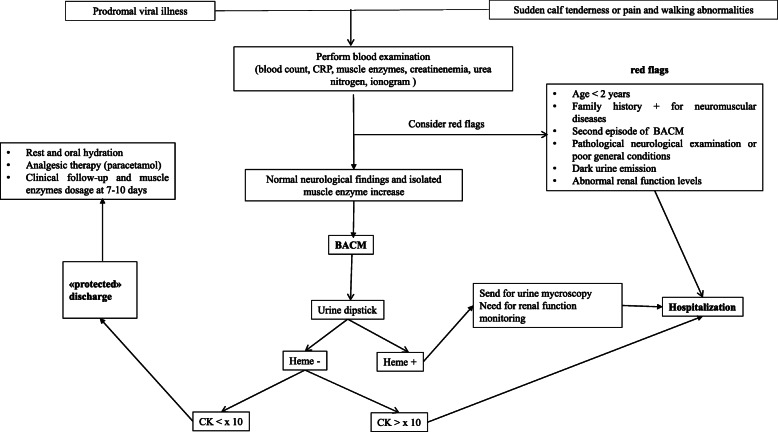
clinical management pathway for BACM in pediatric Emergency Department

In conclusion, in this study we provide the largest case series of patients presenting with BACM condition at 2 pediatric emergency departments. Our data emphasize the benign clinical course of BACM condition in order to avoid unnecessary investigations and hospitalization which lead to a waste of resources. In order to support physicians who are not familiar with BACM and to rationalize health resources allocation, we provide a diagnostic pathway for patients presenting with BACM in pediatric ED **(**Fig. 3**).**

Within the flow chart, some red flags must be carefully considered by clinicians since they represent clinical findings suggesting alternative severe diagnosis or elements which need different clinical management.

In patients with at least one red flag, further diagnostic exams should be performed, according to specific patient’s history and clinical picture (i.e., urinary organic acids, molecular tests, neurophysiological study).

In the absence of a proven method of predicting the risk of acute renal failure, we propose that patients with mild symptoms, normal urine dipstick and serum CK levels less than 10X normal values have to be considered low risk patients. They may be discharged from ED and treated as outpatients with oral hydration, analgesics and rest.

However, a clinical and laboratory follow-up within 10–15 days is recommended to confirm the diagnosis and the benign prognosis.

## Data Availability

The datasets used and/or analyzed during the current study are available from the corresponding author on reasonable request.

## Abbreviations

BACM: benign acute childhood myositis
CK: creatine kinase
ED: Emergency Department
